# Assessing risk of stroke after idiopathic sudden sensorineural hearing loss using data from general practice

**DOI:** 10.1038/s41598-024-59934-3

**Published:** 2024-05-01

**Authors:** Fieke K. Oussoren, Tjard R. Schermer, Leonie R. Horn, Roeland B. van Leeuwen, Tjasse D. Bruintjes

**Affiliations:** 1 Department of Otorhinolaryngology, Leiden University Medical Center, P.O. Box 9600, 2300 RC Leiden, The Netherlands; 2 Apeldoorn Dizziness Centre, Gelre Hospitals, Apeldoorn, The Netherlands; 3 Department of Primary and Community Care, Radboudumc Research Institute for Medical Innovation, Radboud University Medical Center, P.O. Box 9101, 6500 HB Nijmegen, The Netherlands

**Keywords:** Idiopathic sudden sensorineural hearing loss, Stroke, General practice, Cardiovascular risk, Medical research, Neurology, Risk factors, Signs and symptoms

## Abstract

The cause of sudden sensorineural hearing loss (SSNHL) remains unknown in a significant number of cases, but vascular involvement in its pathophysiology has been proposed. Our study aimed to assess the incidence of stroke following idiopathic SSNHL (iSSNHL) and to evaluate associated cardiovascular risk factors and comorbidities. We extracted electronic medical record data from iSSNHL patients aged ≥ 50 years retrospectively from 84 general practices. Patients were matched for age, sex and general practice in a 1:4 ratio to controls. Primary outcome was the 5-years stroke risk following iSSNHL diagnosis. 480 iSSNHL cases could be matched to 1911 controls. The hazard ratio for iSSNHL compared with controls was 1.25 (95%CI 0.50–3.27; *P* = 0.646) for CVA (cerebrovascular accident) alone and 0.92 (95% CI 0.50–1.71; *P* = 0.804) for CVA and TIA (transient ischemic attack) combined. The hazard ratio for the interaction term between iSSNHL and age ≥ 60 years was 4.84 (95% CI 1.02–23.05; *P* = 0.048) for CVA and TIA combined. Patients with iSSNHL used antihypertensives and beta-blocking agents more frequently than controls (*P* = 0.006 and *P* = 0.022, respectively). In conclusion, no overall significant difference in the risk of stroke was observed, but the hazard ratio for stroke increased in iSSNHL patients aged 60 and older, suggesting potential vascular involvement in older subjects presenting with sudden sensorineural hearing loss.

## Introduction

The exact cause of sudden sensorineural hearing loss (SSNHL) remains unknown in a significant number of cases. Suspected causes for SSNHL include infectious diseases, particularly of viral origin, auto-immune disease, vascular insufficiency, and neoplasms. The current therapy with high dose corticosteroids is based on the assumption that sudden deafness is caused by inflammation secondary to viral infection. However, the clinical benefit of this therapy remains questionable, with hearing recovery reported in only 30–50% of cases^1^.

In 2008, Lin et al. proposed vascular involvement in the pathophysiology of acute hearing loss^2^. They observed a higher risk of stroke following sudden sensorineural hearing loss compared to a control group. Various other researchers have reported higher incidence rates of a cerebrovascular accident (CVA) in patients who experienced SSNHL compared to the general population^3–7^. These authors hypothesized that acute hearing loss may serve as a prodrome to a CVA, potentially warranting targeted therapy with anticoagulants^3–7^.

One of the primary concerns with this hypothesis is that available literature relies on data sources from non-medical organizations, such as health insurance databases^6,8^. This may introduce inclusion bias, wherein patients with sensorineural hearing loss who did not meet the global criteria could still be assigned the administrative billing code for SSNHL because it appeared to be the most appropriate choice. Also, when utilizing these databases, the available information regarding cardiovascular comorbidity and risk factors, its treatments, and their duration is limited. Consequently, establishing associations between cardiovascular involvement in SSNHL and the preventive impact of appropriate therapy is challenging.

The objectives of this retrospective study were to analyze cardiovascular comorbidity and cardiovascular risk factors at the time of an idiopathic SSNHL event, and to assess the subsequent risk of a CVA or transient ischemic attack (TIA) in these patients. We accomplished this using data extracted from electronic health records from general practices in the Netherlands.

## Methods

### Study population

This is a retrospective matched case–control study based on de-identified electronic medical records from the Radboud University Medical Center primary care database, which contains data from 84 general practices in the Netherlands. Health records spanning from January 1st 2011 until December 31st 2021 were analyzed. Cases included individuals of 18 years or older who experienced an episode of idiopathic SSNHL (iSSNHL) within this study period. Cases were compared to a control cohort without iSSNHL. Subjects with a documented medical history of SSNHL before the start of the study date were excluded.

The diagnosis of iSSNHL was established by using ICD-10 codes (International Classification of Diseases and Related Health Problems, 10th revision) as well as ICPC-1 codes (International Classification of Primary Care). In the ICD-10 system, sudden hearing loss is defined by the code H91.2. As the ICPC coding system lacks a specific code for sudden hearing loss, cases were identified using their episode title, the presence of an ear related issue marked with “H”, and through manual searching free text fields. The latter could include descriptions entered by the GPs such as “sudden hearing loss”, “sudden deafness”, or equivalent synonyms.

Controls were randomly selected from the same primary care database and were matched to cases based on age, sex, general practice, and a similar registration year at the GP practice, with a matching ratio of 1 iSSNHL case to 4 controls. Because all subjects with documented iSSNHL in their general practice medical record were selected as potential cases for the study, controls could not have had iSSNHL at any point during their follow-up until December 31, 2021.

### Outcomes

The primary outcome was the difference in incidence of a CVA or a transient ischemic attack (TIA) within a 5-year follow-up period between the patients with iSSNHL and the controls. A secondary outcome was the prevalence of cardiovascular comorbidity between patients with iSSNHL and controls. Information on the following cardiovascular comorbidities and their corresponding ICPC codes was gathered from the general practice medical records: arterial aneurysm (K99.01), atrial fibrillation (K78), CVA (K90), congestive heart failure (K77), hypertension (K74-K87), ischemic heart disease (K74-K76), lipid spectrum disorders (T93), peripheral occlusive arterial disease (PAOD) (K91, K92), thrombosis (K93, K94.01), and TIA (K89).

Other secondary outcomes were differences between iSSNHL cases and controls in terms of cardiovascular risk factors: smoking (P17), diabetes mellitus (T90), Body Mass Index (BMI), systolic and diastolic blood pressure, and laboratory results for fasting glucose, glycated hemoglobin (HbA1c), low-density lipoprotein (LDL) cholesterol, high-density lipoprotein (HDL) cholesterol and triglycerides. These data were extracted from the nearest available visit or measurements within a specified time interval before or after the iSSNHL episode data or (for the matched controls) the selected control date. The maximum interval between disease onset and the measurement or lab results we allowed was defined in accordance with the Dutch guideline for cardiovascular risk management^9^. Final secondary outcomes were differences in therapy administered for the treatment of cardiovascular comorbidity and cardiovascular risk factors. These were identified using ATC (Anatomical Therapeutic Chemicals) codes for various drug therapies: antidiabetics (A10), anticoagulants (B01), antihypertensives (C02), diuretics (C03), beta-blocking agents (C07), calcium channel blockers (C08), agents acting on the renin-angiotensin system (C09), lipid modifying agents (C10), and organic nitrates (C01DA).

#### Statistical analysis

Categorical variables are displayed as numbers and percentages, while continuous variables are reported as means and standard deviations (SD) for normally distributed data or as median and interquartile ranges (IQR) for skewed data. Population characteristics and the presence of cardiovascular comorbidity and risk factors and their therapies were compared between cases and controls using Chi-square tests for categorical variables and independent sample t-tests for continuous variables.

The incidence of CVA events alone and of CVA or TIA events combined was analyzed using Cox proportional hazard regression models. Initially a univariable Cox model was used with a 5-year follow-up period. If patients did not complete the full five-year follow-up, they were censored on the time of death or departure from the general practice. A multivariable Cox model was subsequently used to analyze the difference between both groups while accounting for potential confounding variables (i.e., cardiovascular comorbidity and cardiovascular risk factors). Finally, to specifically address the effect of age, the study population was divided into two subgroups: subjects aged 60 years and older and subjects younger than 60, respectively. The cut-off value for older age was set on 60 years as this was the median age in the entire cohort. In a separate Cox model an interaction term (age subgroup * iSSNHL) was entered to assess potential effect modification of the risk of CVA or TIA due to iSSNHL by (older) age. Results of all Cox models are presented as hazard ratios (HRs) with 95% confidence intervals (CI).

The data were analyzed using SPSS (IBM Corp. Released 2019. IBM SPSS Statistics for Windows, Version 27.0. Armonk, NY: IBM CorpS). A significance level of a two-tailed *P* value < 0.05 was used to define statistical significance.

### Ethics approval and consent to participate

All methods applied were carried out in accordance with relevant guidelines and regulations following the Dutch Code of Conduct for Health Research (see https://www.coreon.org/wp-content/uploads/2023/06/Code-of-Conduct-for-Health-Research-2022.pdf). All relevant protocols were approved by the Radboud University Medical Center ethics review board (METC Oost-Nederland). Obtaining informed consent from subjects and/or their legal guardian(s) for publication of de-identified medical record information in an online open-access publication was waived by the METC Oost-Nederland (file number: 2020–6871).

## Results

### Selection of iSSNHL cases and controls

**Figure 1:**
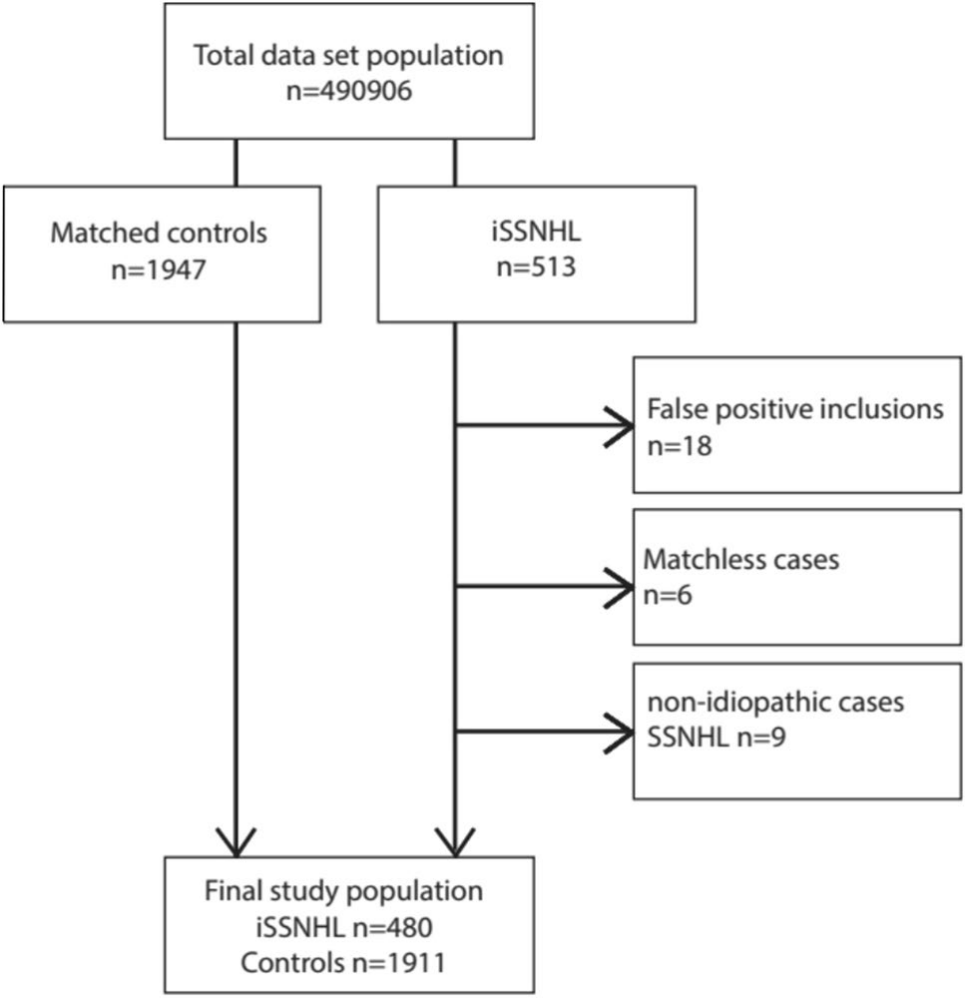
Selection of cases and matched controls. The figure illustrates the inclusion process of 480 eligible cases with idiopathic sudden sensorineural hearing loss and 1911 matched controls from a total of 490,906 subjects in the primary care database containing coded health record data from 84 general practices in the Nijmegen region, the Netherlands. iSSNHL: idiopathic sudden sensorineural hearing loss.

A total of 513 sudden deafness cases were identified. Among these, 18 cases had incorrectly received a positive diagnosis of SSNHL, as became clear from the free text fields. Additionally, nine cases were excluded as they were non-idiopathic cases of SSNHL. This resulted in 480 iSSNHL cases that could be matched to controls. For 9 of these iSSNHL cases only three (instead of the intended four) suitable controls could be identified, while all other cases were matched with 4 controls each. This resulted in a total of 1911 controls. Figure 1 illustrates the selection process.

### Baseline characteristics

Table 1 provides an overview of the patient characteristics, cardiovascular comorbidity and cardiovascular risk factors for the iSSNHL cases and their matched controls. Atrial fibrillation was the only cardiovascular comorbidity that appeared significantly more prevalent in the iSSNHL cohort than in the control cohort (*P* = 0.037). Angina pectoris, congestive heart failure, diabetes, hypertension, obesity, PAOD, thrombosis and history of a CVA were more frequently seen in patients experiencing iSSNHL, but these apparent differences in prevalence did not reach statistical significance. Importantly, Table 1 also shows the substantial amount of missing data for the cardiovascular risk factors in both cohorts.

**Table 1:** Patient characteristics, cardiovascular comorbidity and cardiovascular risk factors of patients with idiopathic sudden sensorineural hearing loss and matched controls.

	iSSNHL (n = 480)	Controls (n = 1911)	*P* value
Demographic characteristics			
Age (SD)	59.1 (14.3)	59.0 (14.2)	0.846
Sex, n (%) males	268 (55.8)	(55.6)	0.959
Cardiovascular comorbidity			
Aneurysm ^a^	4 (0.8)	19 (1.0)	1.00
Angina pectoris	24 (5)	82 (4.3)	0.535
Atrial fibrillation	28 (5.8)	69 (3.6)	0.037
Congestive heart failure	11 (2.3)	28 (1.5)	0.225
Ischemic heart disease ^j^	29 (6.0)	130 (6.8)	0.609
PAOD ^b k^	33 (6.9)	109 (5.7)	0.332
Thrombosis ^m^	10 (2.0)	29 (1.9)	0.312
TIA^n o^	11 (2.3)	62 (3.2)	0.372
CVA^n o^	18 (3.8)	56 (2.9)	0.376
Cardiovascular risk factors			
BMI ^i^ (kg/m^2^)			0.752
< 24.9	72 (45.0)	265 (45.3)	
25–29.9	43 (26.8)	162 (27.6)	
30–39.9	42 (26.2)	155 (26.5)	
> 39.9	3 (1.9)	9 (1.5)	
Number of missings	320	326	
Smoking ^b l^	36 (7.5)	162 (8.5)	0.518
Number of missings	444	1,749	
Diabetes Mellitus ^b c^	46 (9.6)	168 (8.8)	0.592
Venous glucose ^d^ (mean (SD) mmol/l)	6.0 (1.4)	6.1 (1.5)	0.522
Number of missings	356 (74.2)	1483 (77.6)	
HbA1c ^e^ (mean (SD) mmol/mol)	50.1 (12.1)	50.0 (13.2)	0.959
Number of missings	440 (90.7)	1732 (90.6)	
Dyslipidemia^b f^	59 (12.3)	239 (12.5)	0.939
LDL cholesterol ^g^(mean (SD) mmol/l)	3.24 (1.0)	3.13 (1.0)	0.116
Number of missings	156 (32.5)	687 (35.9)	
Non-HDL cholesterol ^g^(mean (SD) mmol/l)	3.7 (1.1)	3.8 (1.1)	0.343
Number of missings	438 (91.3)	1732 (90.6)	
Triglycerides ^g^(mean (SD) mmol/l)	1.4 (0.8)	1.6 (0.9)	0.051
Number of missings	223 (46.5)	939 (49.1)	
Hypertension ^b h^	148 (30.8)	531 (27.8)	0.193
Systolic blood pressure ^i^(mean (SD) mmHg)	139 (18.1)	137 (18.8)	0.138
Diastolic blood pressure^i^(mean (SD) mmHg)	80 (10.1)	79 (10.7)	0.239
Number of missings	276 (57.5)	1214 (63.5)	

Figures are n (%) unless stated otherwise.

BMI, body mass index; CVA, cerebrovascular accident; HbA1c, hemoglobin A1c; HDL, high-density lipoprotein; iSSNHL, idiopathic sudden sensorineural hearing loss; LDL, low-density lipoprotein; PAOD, peripheral arterial occlusive disease; SD, standard deviation; TIA, transient ischemic attack.

^a^Fisher exact test for difference in aneurysm prevalence. ^b^Based on notifications in patients’ medical records. ^c^Diabetes mellitus type 1 or type 2. ^d^Measured within half a year interval (180 days) before or after episode of ISSNHL or control date. ^e^Measured within two-thirds years interval (210 days) before or after episode of iSSNHL or control date. ^f^Dyslipidaemia includes fat metabolism disorder(s), hypercholesterolemia, hypertriglyceridemia, mixed hyperlipidaemia, and familial hypercholesterolemia. ^g^Measured within one-and-a-half-years interval (731 days) before or after episode of iSSNHL or control date. ^h^Hypertension includes increased blood pressure, essential hypertension without organ damage, and hypertension with organ damage/secondary hypertension. ^i^Measured within one year interval (365 days) before or after episode of iSSNHL or control date. ^j^Ischemic heart disease includes acute myocardial infarction, other/chronic ischemic heart disease, coronary artery sclerosis, previous myocardial infarction (> 4 weeks ago). ^k^Peripheral Arterial Occlusive Disease includes atherosclerosis, intermittent claudication, Raynaud's syndrome, Buerger's disease, and other disease(s) peripheral arteries. ^l^Former and current smokers. ^m^Thrombosis includes deep vein thrombosis and lung embolism. ^n^TIAs and CVAs up to the iSSNHL or control date. ^o^CVA includes subarachnoid haemorrhage, intracerebral haemorrhage, and cerebral infarction.

### Management of cardiovascular comorbidity and cardiovascular risk factors

Table 2 presents an overview of the pharmacotherapy for cardiovascular comorbidity and risk factors. A significantly higher proportion of patients with iSSNHL used medication for cardiovascular comorbidities. A statistically significant higher proportion of iSSNHL patients used antihypertensive drugs and beta-blocking agents. The percentage of patients using other therapeutic agents was comparable between both cohorts.

**Table 2:** Treatment of cardiovascular comorbidity and cardiovascular risk factors.

	i**SSNHL** (n = 480)	**Controls** (n = 1,911)	**P-value**
No medication use, n (%)	217 (45.2)	984 (51.5)	0.016
Medication use, n (%)	263 (44.8)	927 (48.5)	
Anticoagulants	130 (27.0)	459 (24.0)	0.175
Antidiabetics	40 (8.3)	160 (8.4)	1.000
Antihypertensives	**15 (3.1)**	**23 (1.2)**	**0.006**
Diuretics	113(23.5)	419 (21.9)	0.425
Beta-blocking agents	**153 (31.9)**	**508 (26.6)**	**0.022**
Calcium channel blockers	91 (19.0)	326 (17.1)	0.313
Agents acting on renin angiotensin system	137 (28.5)	565 (29.6)	0.695
Lipid modifying agents	164 (34.2)	600 (31.4)	0.250
Organic nitrates	44 (9.2)	174 (9.1)	0.929

### Risk of CVA and TIA

During the 5-year follow-up, a total of 30 CVAs and 31 TIAs were recorded within the entire study population. Five cases of a CVA (1.0%) and 8 cases of transient ischemia (1.7%) occurred in the iSSNHL cohort, compared to 25 cases of CVA (1.3%) and 23 cases of transient ischemia (1.2%) in the control cohort.

**Figure 2:**
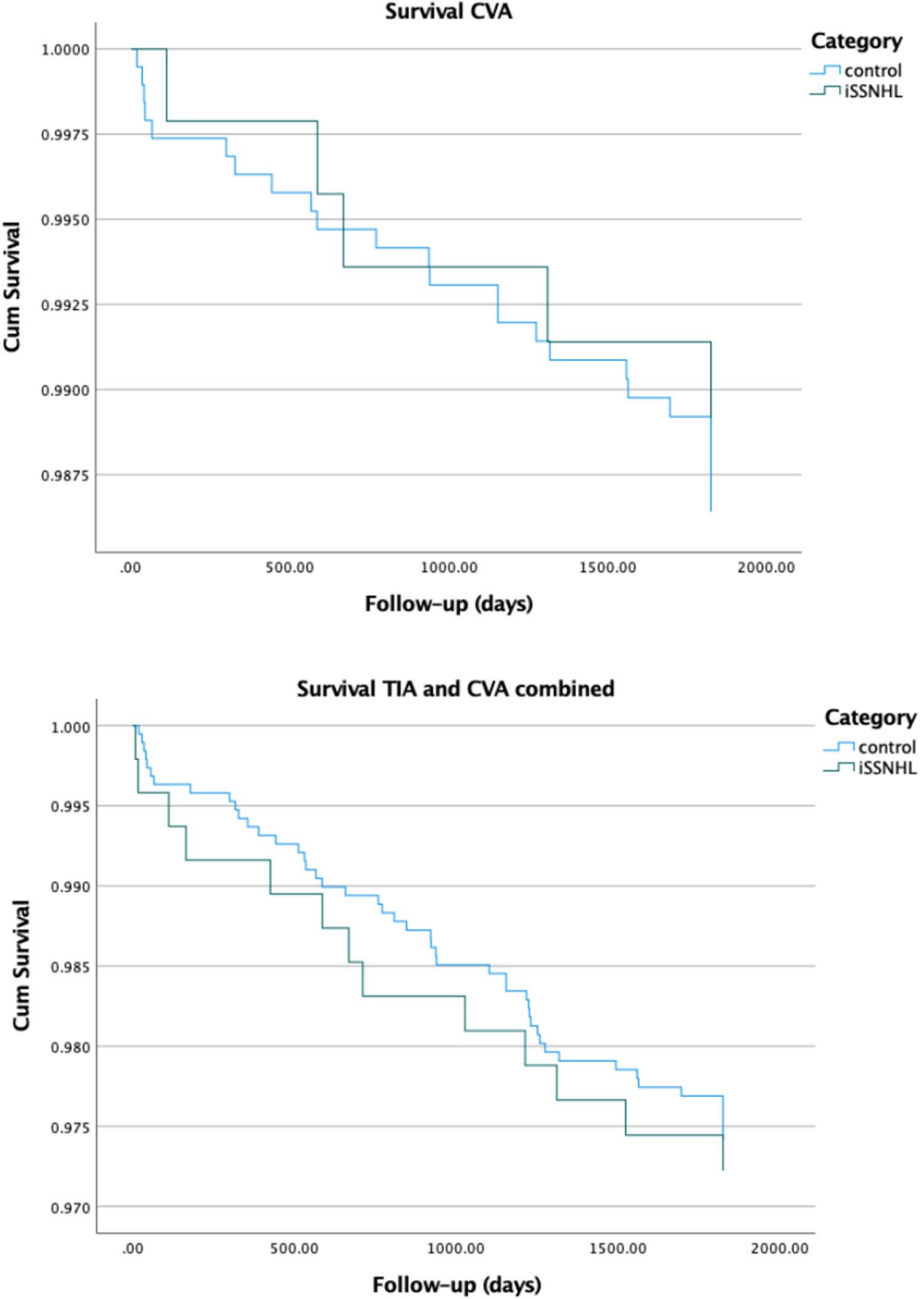
Cox regression survival curves for CVA alone (panel a), and CVA and TIA combined (panel b) in the two cohorts during the 5-year follow-up period.

In the univariable Cox regression models, iSSNHL alone did not show a statistically significant hazard ratio for CVA or for CVA and TIA combined (see Fig. 2 and Table 3); the hazard ratio for iSSNHL compared with controls was 1.25 (95% CI 0.50– 3.27; *P* = 0.646) for CVA alone and 0.92 (95% CI 0.50–1.71; *P* = 0.804) for CVA and TIA combined. Only age, cardiac disease, hypertension and diabetes univariately showed significant associations with the hazard of CVA.

**Table 3:** Hazard ratios from univariable and multivariable Cox regression analyses for occurrence of CVA and CVA or TIA combined within 5 years follow up.

*Characteristic*	Univariable analysis		Multivariable analysis	
	Hazard ratio (95%CI)	*P* value	Hazard ratio (95%CI)	*P* value
Outcome: CVA				
iSSNHL	1.25 (0.50, 3.27)	0.646		
Age	**1.10 (1.07, 1.14)**	** < 0.01**	**1.06 (1.04, 1.09)**	** < 0.001**
Angina Pectoris	0.29 (0.10, 0.82)	0.020		
Aneurysm	0.26 (0.04, 1.92)	0.188		
Atrial fibrillation	0.56 (0.13, 2.33)	0.422		
Cardiac disease	**0.27 (0.11, 0.67)**	**0.004**	0.67 (0.33, 1.35)	0.258
Congestive heart failure	0.40 (0.06, 2.95)	0.370		
CVA ^a^	0.88 (0.12, 6.43)	0.896		
Diabetes mellitus	0.48 (0.18, 1.25)	0.130		
Dyslipidaemia	0.93 (0.32, 2.65)	0.882		
Hypertension	**0.34 (0.17, 0.70)**	**0.003**	0.68 (0.41, 1.15)	0.151
Obesity	1.07 (0.15, 7.83)	0.949		
PAOD	0.38 (0.13, 1.10)	0.074		
Smoking	2.61 (0.35, 19.1)	0.346		
Thrombosis	**0.22 (0.05, 0.92)**	**0.038**	0.80 (0.19, 3.34)	0.760
Outcome: CVA or TIA				
iSSNHL	0.92 (0.50, 1.71)	0.804		
Age	**1.07 (1.05, 1.10)**	** < 0.01**	**1.06 (1.04, 1.10)**	** < 0.001**
Angina Pectoris	0.50 (0.20, 1.24	0.136		
Aneurysm	0.55 (0.08, 3.95)	0.550		
Atrial fibrillation	0.45 (0.18, 1.12)	0.084		
Cardiac disease	**0.35 (0.18, 0.69)**	**0.002**	0.68 (0.34, 1.37)	0.282
Congestive heart failure	0.84 (0.12, 6.10)	0.865		
CVA ^a^	1.81 (0.25, 13.1)	0.555		
Diabetes mellitus	**0.43 (0.23, 0.84)**	**0.013**	0.77 (0.39, 1.50)	0.437
Dyslipidaemia	1.30 (0.56, 3.02)	0.540		
Hypertension	**0.40 (0.24, 0.67)**	** < 0.001**	0.70 (0.41, 1.18)	0.180
Obesity	2.21 (0.31, 15.96)	0.431		
PAOD	0.54 (0.23, 1.26)	0.156		
Smoking	1.28 (0.47, 3.53)	0.633		
Thrombosis	0.46 (0.11, 1.89)	0.281		

CVA, cerebrovascular accident; iSSNHL, idiopathic Sudden Sensorineural Hearing Loss; PAOD, Peripheral Aortic Occlusive Disease; TIA, transient ischemic attack; Statistically significant associations are marked bold**.**

^a^Before the iSSNHL episode or the selected starting date for the 5-year follow-up in the controls (i.e., history of CVA).

In the multivariable Cox regression models, increased age (defined as 1 year older) showed a slightly increased hazard for an ischemic cerebral event, with hazard ratios of 1.06 (95% CI 1.04–1.10; *P* < 0.001) and 1.06 (95% CI 1.04–1.10; *P* < 0.001) for CVA alone and CVA and TIA combined, respectively (Table 3). iSSNHL nor any of the cardiovascular comorbidities or cardiovascular risk factors showed statistically significant hazard ratios in the two multivariate Cox models.

In an additional Cox model we further explored the role of older age (i.e., above 60 years) as an effect modifier when considering iSSNHL as a potential risk factor for CVA or TIA. This model showed that the hazard ratio for the interaction term between iSSNHL and age category was statistically significant at 4.84 (95% CI 1.02– 23.05; *P* = 0.048) (Table 4). This indicates that in the subgroup aged ≥ 60 iSSNHL increased the risk of CVA or TIA while this was not the case in the subgroup aged < 60.

**Table 4:** Hazard ratios from the Cox regression model for occurrence of a CVA or TIA with iSSNHL, age category (i.e. < 60 or ≥ 60 years), and the interaction term of these two variables.

	Hazard ratio	95%CI		*P*
		Lower	Upper	
iSSNHL	0.25	0.06	1.01	0.051
Age ≥ 60	2.11	0.65	6.86	0.214
Age ≥ 60 * iSSNHL	**4.84**	**1.02**	**23.05**	**0.048**

CI, Confidence interval; iSSNHL, idiopathic sudden sensorineural hearing loss; Statistically significant associations are marked bold.

## Discussion

 In this retrospective, matched case–control study using data from general practices we observed no overall increased hazard rate for CVA or TIA in the 5 year follow-up after an episode of iSSNHL when compared to controls. The only cardiovascular risk factor that was significantly more prevalent in the iSSNHL cohort was atrial fibrillation. Patients with iSSNHL seemed to use more medication to treat cardiovascular comorbidity. Beta-blocking agents and antihypertensives, not belonging to the group of diuretics, calcium channel blockers or agents acting on renin angiotensin system, were significantly more used by patients with iSSNHL than by controls. Age was related to increased risk of CVA or TIA, and in subjects aged 60 years and older iSSNHL appeared to increase the risk of CVA or TIA compared to younger subjects. The latter observation, which has not been described in other research already done on this topic, may be explained as follows. Multiple factors may be involved in SSNHL, including an inflammatory component (viral) and/or a vascular component. In younger people there are few vascular risk factors and a vascular genesis of SSNHL is unlikely. In elderly subjects, increasing vascular risk factors and age itself increase the risk of stroke, and thus the chance that iSSNHL is a vascular incident. Having a vascular incident in itself increases the risk of a (new) stroke. So the fact that more strokes occurred in the iSSNHL group compared to the control group at age 60 + may indicate that their iSSNHL was of a vascular nature. A possible clinical implication is to consider starting anticoagulation treatment in patients aged 60 + with an iSSNHL.

Since 2008, seven papers have investigated the risk of CVA following sudden sensorineural hearing loss, as several authors previously proposed being vascularly compromised as a contributing factor in the pathophysiology of iSSNHL^2–4,6,7,10^. The potential vascular involvement in the pathophysiology of SSNHL could have a significant impact on the treatment approach of iSSNHL. Currently, acute hearing loss is primarily treated with corticosteroids, based on the assumption of inflammation secondary to viral infection as the underlying mechanism (or auto-immune). To the best of our knowledge, the consideration of cardiovascular risk management, including anticoagulant therapy, as a therapeutic approach of acute hearing loss is only practiced in Germany^11^.

In 2020, Lammers et al. conducted a meta-analysis comparing the results of studies investigating the risk of a CVA after SSNHL^12^. Due to significant heterogeneity in results they could only include 3 articles in the meta-analysis. This resulted in a hazard ratio of 1.42 (95% CI 1.15–1.75) for CVA compared with controls without sudden hearing loss. In contrast, our study did not find an overall increased risk of CVA following iSSNHL. The main issue with the outcome of the included studies in the meta-analysis is their study design. Most articles relied on non-medical data from national health insurance databases, where all patients with the same billing code were assumed to have had sudden sensorineural hearing loss. However, just like in our study the degree of hearing loss could not be retrieved and patients who may not have met the criteria for iSSNHL may nonetheless have received a billing code for iSSNHL for administrative reasons. In addition, data concerning cardiovascular comorbidity, cardiovascular risk factors and related treatment may not always be that accurate in such a database.

Similar to our findings, Ciorba et al., the only previous European study investigating the incidence of CVA in an iSSNHL cohort, did not report an association between iSSHNL and CVA^3^. However, the study design of this study also had some limitations. The authors compared the incidence of CVA in patients with iSSNHL to the expected incidence of CVA based on population estimates. Also, the cohorts were not matched for age or other potential confounders.

Our study’s primary strength lies in the use of medical record data derived from general practices. By using data from this source rather than on the basis of national health insurance data, the study was designed to reduce inaccuracies in the diagnosis of sudden hearing loss. In the Netherlands, it is common for patients with iSSNHL to see their GP before receiving an emergency referral to an ENT outpatient clinic if there is suspicion of sudden deafness. Consequently, our cohort offers an estimation of the incidence of iSSNHL in the entire population. In the Dutch healthcare system, GPs are responsible for cardiovascular risk management. Thus, we could compare cardiovascular risk factors in subjects with iSSNHL to the matched controls. Cardiovascular risk factors nor cardiovascular comorbidity were more prevalent in the iSSNHL cohort than in the control cohort. Objective measurements of glucose, blood pressure and lipid levels also did not significantly differ between both cohorts. It is noteworthy that for most of the cardiovascular risk factors their presence appeared to reduce the hazard rates of a CVA or TIA, which may seem unusual given the hypothesis of vascular involvement in iSSNHL. However, factors such as older age, obesity, and smoking, which cannot (easily) be altered by medical treatment, showed an increased risk of CVA or TIA, although not all were statistically significant. This suggests that cardiovascular risk management of modifiable cardiovascular risk factors effectively reduces the incidence of a CVA or TIA, which aligns with the desired effect of these therapies. Our finding that more patients with iSSNHL used medication to treat cardiovascular risk factors aligns with this suggestion.

In this cohort, age was the sole risk factor that significantly increased the risk of a CVA or TIA. While increasing age did not appear to affect the overall risk of these events differently in subjects with and without iSSNHL, subjects with iSSNHL aged 60 or older did have a significantly higher hazard ratio for a CVA or TIA than controls of a similar age. This suggests that older individuals with iSSNHL may have a greater a CVA risk compared to both younger individuals with iSSNHL and older individuals without iSSNHL.

The main limitation of this study is that we could not access audiometry results. Consequently, we relied on the GPs’ ability to diagnose SSNHL accurately. Fortunately, GPs in the Netherlands probably adhere to the Dutch medical guidelines for acute hearing loss, which is based on worldwide accepted criteria for SSNHL. Despite the extensive dataset comprising almost half a million individuals, the overall incidence of CVAs and TIAs in the entire cohort was rather low. Therefore, conclusions drawn from our analyses should be interpreted with caution.

## Conclusions

In our study we did not find an overall statistically significant difference in the risk of a CVA or CVA and TIA combined between subjects with iSSNHL and matched controls. However, the hazard ratio for a CVA or TIA was increased in subjects with iSSNHL aged 60 and older compared to younger subjects with iSSNHL and to older subjects without iSSNHL. This suggests that iSSNHL could have vascular involvement in older subjects, but further research is needed to confirm this observation.

## Data Availability

The dataset used for the current study is available from the corresponding author.

## Abbreviations

BMI: Body Mass Index
CI: Confidence interval
CVA: Cerebrovascular accident
ENT: Ear, nose, and throat
GP: General practitioner
HbA1c: Glycated hemoglobin
HDL: High-density lipoprotein
HR: Hazard ratios
ICD: International Classification of Diseases and Related Health Problems
ICPC: International Classification of Primary Care
iSSNHL: Idiopathic sudden sensorineural hearing loss
LDL: Low-density lipoprotein
PAOD: Peripheral arterial occlusive disease
SD: Standard deviation
SSNHL: Sudden sensorineural hearing loss
TIA: Transient ischemic attack
